# An Unexpected Complication of Gastroenteritis

**DOI:** 10.7759/cureus.89556

**Published:** 2025-08-07

**Authors:** Jolan Grignard, Kevin Silber, Heloise Paccaud, Guérisse Fabien

**Affiliations:** 1 Department of Emergency Medicine, Centre Hospitalier Universitaire de Charleroi, Lodelinsart, BEL; 2 Department of Emergency Medicine, Erasmus Hospital, Bruxelles, BEL

**Keywords:** acute chest pain diagnosis, bacterial gastroenteritis, campylobacter jejuni, myocarditis, pericarditis

## Abstract

A 37-year-old man, previously healthy, presented to the emergency department with retrosternal chest pain for 24 hours, in the context of watery diarrhea (five to six times a day), abdominal pain, and fever evolving over four days. Following medical assessment, a diagnosis of myopericarditis due to *Campylobacter jejuni* infection was made. This complication of *C. jejuni* infection is rare and poorly described, but given its rising incidence, increased vigilance is necessary. The affinity of *C. jejuni* for the myocardium and pericardium remains unclear. The underlying mechanisms are still under investigation, although several hypotheses are emerging. Its insidious presentation and generally favorable clinical course make it an entity often underdiagnosed and widely unrecognized. This case report aims to highlight the existence of this rare complication (0.4%) to prevent diagnostic errors and iatrogenic complications (potentially harmful coronary angiographies or fibrinolysis) that may result from it.

## Introduction

*Campylobacter jejuni* are Gram-negative spiral bacteria and the most common cause of bacterial gastroenteritis in developed countries. Patients with *C. jejuni* infection experience acute watery or bloody diarrhea, fever, weight loss, and cramps that last, on average, six days. The incidence and prevalence of campylobacteriosis have increased in both developed and developing countries over 2005-2015 [1].

Myopericarditis (pericarditis with concurrent myocardial involvement) is rare, accounting for 0.4% of cases of *C. jejuni* infection [2]. Because of the rising number of cases and percentage of acute gastroenteritis due to *C. Jejuni* compared with other pathogens, increased vigilance is necessary [1]. Myopericarditis manifests as pleuritic chest pain, pericardial friction rub on auscultation, pericardial effusion, and abnormalities on ECG (diffuse ST-segment elevation and PR-segment depression). It may sometimes be accompanied by dyspnea or palpitations, but can also present without any typical findings. In this case, myopericarditis was confirmed using the criteria of pericarditis, cardiac biomarkers, and echocardiography. It has been hypothesized that invasion of the cardiac tissue, bacterial exotoxins, circulating immune complexes, and cytotoxic T cells are involved [1]. Usually, myopericarditis resolves spontaneously, with or without any targeted treatment. However, it can lead to hospitalization in the intensive care unit or even death due to severe complications such as cardiac failure, cardiac tamponade, or arrhythmias.

This case report describes a previously healthy 37-year-old man who developed myopericarditis shortly after *C. jejuni* gastroenteritis. We aim to highlight key clinical “red flags” and outline a pragmatic, evidence-based approach that minimizes diagnostic errors and iatrogenic complications (unnecessary invasive coronary angiographies, potentially harmful antithrombotic therapies).

## Case presentation

A 37-year-old man presented to the Department of Emergency of Charleroi University Hospital with ongoing chest pain of one-day duration. The pain was retrosternal, crampy, constant, and radiating to the left arm. It worsened in the supine position, reaching 8/10 on the visual analog scale. His past history was significant for fever, watery diarrhea (five to six times a day), and dyspnea on exertion that lasted for four days before the onset of the current chest pain episode. The patient had undergone surgical treatment for right carpal tunnel syndrome and inguinal hernia repair. He had no past medical history. The patient was a chronic smoker, occasionally consumed alcohol, did not use drugs, and had no known allergies.

On physical examination, his vital signs were within normal limits with a heart rate of 79 beats/minute, oxygen saturation at room air of 97%, respiratory rate of 21 breaths/minute, blood pressure of 114/67 mmHg, and temperature of 36.9°C. Clinical examination revealed diffuse abdominal tenderness without guarding or rebound tenderness. The patient was eupneic at rest, had no signs of overload, and had a normal cardiorespiratory examination. When asked to bend forward, the chest pain was moderately relieved.

**Figure 1:**
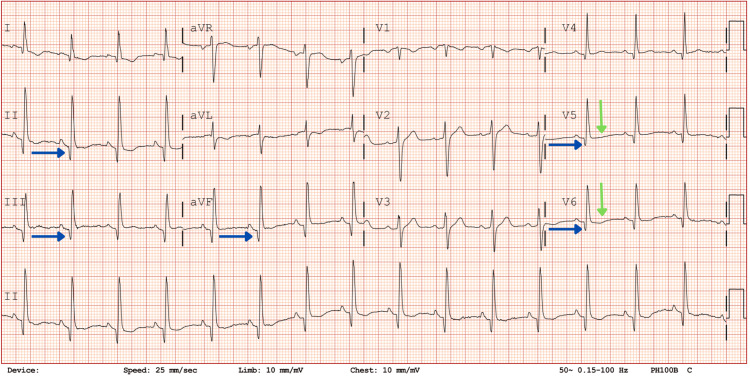
ECG on admission. The blue arrows show Q waves in the inferolateral territory with PR-segment depression. The green arrows show negative T waves in the lateral territory.

**Figure 2:**
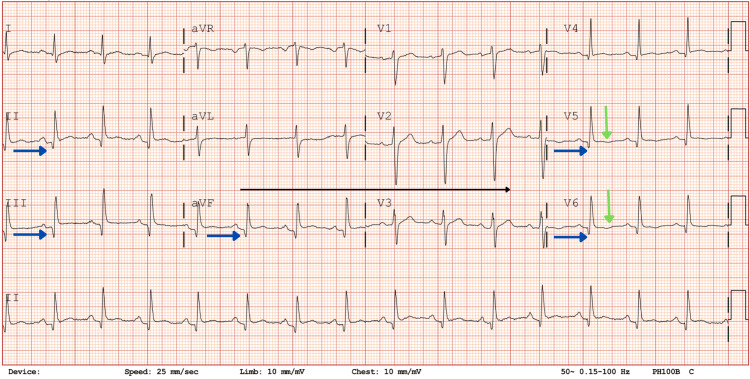
ECG after two hours. The blue arrows show similar Q waves in the inferolateral territory with PR-segment depression. The green arrows show deeper negative T waves in the lateral territory.

ECG showed a regular sinus rhythm of 92 beats/minute associated with Q waves in the inferolateral leads, PR-segment depression, and negative T waves in the lateral leads (Figures 1, 2). Blood tests showed raised inflammatory markers (leukocytes, 11.8 × 10³/mm³; C-reactive protein (CRP), 98 mg/L; fibrinogen, 778 mg/dL), normal renal function and electrolytes, elevated D-dimer at 1,280 ng/mL, mild cholestasis without hyperbilirubinemia, and elevated troponin I at 2.211 ng/mL with creatinine phosphokinase at 363 IU/L (Table 1). Potassium and magnesium were within the normal range. A chest X-ray was normal. Lipase was measured to rule out pancreatitis. Mild liver and pancreatic enzyme elevations were considered associated with systemic inflammation of campylobacteriosis. Autoimmune markers were negative.

**Table 1:** Key laboratory results from admission to discharge.

Test	Day 1	Day 2	Day 5	Reference range
Hemoglobin	14.3	13.1	12	14–18 g/dL (men); 12–16 g/dL (women)
White blood cell count	11.8	7.82	9.45	4.0–10.0 × 10³ cells/mm³
Platelet count	267	264	380	150–400 × 10³ cells/mm³
C-reactive protein	98	-	6	<5 mg/L
Prothrombin time	122%	122%	-	>70%
Creatinine	0.90	0.77	0.77	0.3–0.9 mg/dL
Glomerular filtration rate	94.5	113.1	113.1	>60 mL/minute
Alanine aminotransferase	98	144	110	10–35 U/L
Aspartate aminotransferase	99	138	50	10–35 U/L
Alkaline phosphatase	-	122	104	30–105 IU/L
Gamma-glutamyl transpeptidase	515	514	418	<36 U/L
Lactate dehydrogenase	302	-	212	<250 U/L
Lipase	-	-	246	13–60 U/L
Creatinine kinase	363	-	93	<165 U/L
Troponin I	2.211	2.109	0.046	<0.030 ng/mL
N-terminal pro-B-type natriuretic peptide	-	255	-	<125 pg/mL

Due to chest pain with modified ECG, a transthoracic echocardiography (TTE) was performed, which showed preserved global left ventricular function but mild inferior-basal and infero-medial hypokinesis without any pericardial effusion. The Pulmonary Embolism Rule-Out Criteria were used to exclude pulmonary embolism.

Urine cultures, blood cultures, and viral serology panel (Epstein-Barr virus (EBV)/cytomegalovirus (CMV)/human immunodeficiency virus (HIV)/hepatitis B virus (HBV)/hepatitis C virus (HCV)) were all negative. Stool cultures were also sampled directly on the day of admission in the ED and returned positive on the second day for *C. jejuni*. Other enteric pathogens were negative. The polymerase chain reaction test for SARS-CoV-2 was negative.

The patient was hospitalized in the coronary unit for clinical monitoring. Treatment with aspirin 1g thrice/day, colchicine 0.5 mg twice/day, bisoprolol 2.5 mg half/day, and azithromycin 500 mg once/day for three days was initiated. Bisoprolol was used for rate control and arrhythmia prophylaxis. The use of azithromycin aligns with the local resistance patterns and use habit to treat campylobacteriosis.

Chest pain was rapidly relieved after 24-48 hours of treatment. The patient was hemodynamically stable and did not present with any rhythm disturbance. Biologically, an inflammatory syndrome and a decrease in troponin argued against an acute coronary syndrome. Given the rapidly favorable clinical and biological evolution, the low-risk Global Registry of Acute Coronary Events (GRACE) score, and the high diagnostic suspicion of myopericarditis (and therefore low probability of acute coronary syndrome), a coronary CT angiography was performed rather than a coronary angiography. The examination revealed no coronary lesions. Cardiac MRI was considered but not performed because of the difficulty in obtaining it in an emergency the clarity of the diagnosis. Biopsy was judged too invasive and not necessary in this case.

**Figure 3:**
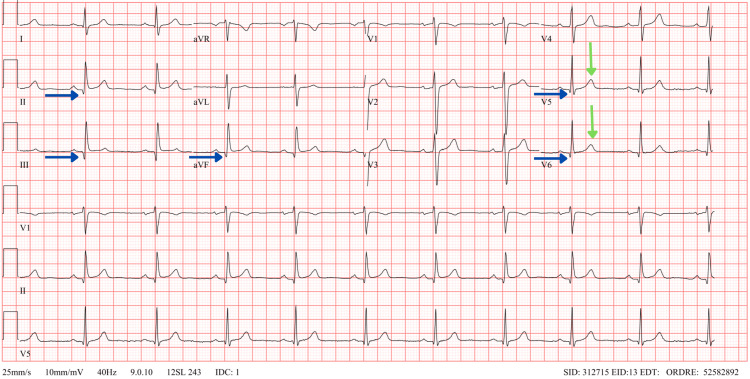
ECG at the four-month follow-up. The blue arrows show attenuated Q waves in the inferolateral territory and resolution of PR-segment depression. The green arrows show resolution of negative T waves in the lateral territory.

The diagnosis of myopericarditis due to *C. jejuni* infection was made. The patient was able to return home after the fifth day of hospitalization with a prohibition of sports for three to six months and a strong recommendation for cardiologic follow-up. Smoking cessation was also heavily encouraged. A normal TTE at one month revealed resolution of cardiac kinetic disturbances, and the four-month follow-up ECG indicated near resolution of ECG abnormalities (Figure 3).

## Discussion

*C. jejuni* is the most common cause of bacterial gastroenteritis or enterocolitis in developed countries, with increasing prevalence [1]. Generally, the illness resolves spontaneously but can be complicated early by local (cholecystitis, peritonitis, urticaria, erythema nodosum, septic aortic pseudoaneurysm) or distant (myocarditis) complications. It can also lead to secondary arthritis or Guillain-Barré syndrome. Myopericarditis affects only 0.4% of cases of *C. jejuni* infection [2]. Cases of myocarditis associated with other *Campylobacter* spp., including *C. coli*, have also been reported [3].

The gastrointestinal manifestations of *C. jejuni* infection are typically diarrhea, abdominal pain, fever, and vomiting. Myopericarditis occurs within a minimal average period of two to five days and presents with variable clinical signs, similar to viral or autoimmune myocarditis. Patients can present with vague symptoms as palpitations, pericardial friction rub on auscultation, and dyspnea, besides the typical pain relieved by trunk flexion. Echocardiography may reveal pericardial effusion or possible contractility disorders, and frequently, ECG reveals diffuse ST-segment elevation and/or PR-segment depression [4].

Myopericarditis is characterized by diffuse inflammation of the myocardium and pericardium. Several categories are responsible for the latter, such as infectious (largely viral, followed by bacterial, and, finally, parasitic), immune/systemic, toxic, hypersensitivity/drug-induced, iatrogenic (post-surgical, post-radiation, etc.), and idiopathic [5].

Young men constitute by far the most affected subpopulation by this condition [4]. Currently, there is no formal explanation for this epidemiological data, but the hormonal pathway remains the most plausible (similar to the majority prevalence of venous thromboembolic disease in women). Indeed, in a study on mice focusing on viral myocarditis, it was highlighted that testosterone would inhibit the local anti-inflammatory response and, conversely, that estrogens would have a protective effect on the myocardium [6].

The pathophysiology of myopericardial lesions related to *Campylobacter* infection has not yet been elucidated. The proposed theories are, on the one hand, a primary direct cardiac attack by the microorganism or its toxins, and, on the other hand, a secondary cardiac attack through an immune-mediated inflammatory cascade [7]. The first hypothesis seems more convincing, primarily due to the temporal component. Indeed, a short interval (<96 hours) between digestive symptoms and the onset of cardiac signs or symptoms argues more for an organism or toxin-mediated mechanism [8], as an immune-induced mechanism requires a minimum of two to three weeks to occur, as observed in Guillain-Barré syndrome through the involvement of cytotoxic T cells [9]. Furthermore, in the hypothesis of a primary attack, the absence of *Campylobacter* detection by PCR (or blood culture) despite clinically florid infections is better explained if bacterial toxins play a predominant role [10]. These elements, however, do not formally exclude the immune-induced component. Additionally, Hessulf et al. suggest that viral co-infection would be a potential causal factor, highlighting the need for serological studies during the patient's assessment, in addition to excluding differential diagnoses of myopericarditis (CMV, EBV, HIV, hepatitis A virus, B19, enterovirus, parechovirus) [11].

Regarding additional investigations, it is recommended to perform routine blood tests, including cardiac and inflammatory markers (troponin, creatine kinase, creatine kinase-MB, CRP, etc.), viral serology panel, and autoantibodies, as well as blood cultures and stool cultures. Finally, ECG remains essential to complement TTE (or transesophageal echocardiography).

Cardiac magnetic resonance is the reference test for confirming myopericarditis when the diagnosis is uncertain or when ventricular dysfunction persists after 48 hours [4]. Typical findings are regional edema and sub-epicardial late gadolinium enhancement in a non-ischemic pattern [4]. Endomyocardial biopsy, the histological gold standard, is reserved for patients with severe, refractory heart failure or life-threatening arrhythmias, owing to its invasiveness and patchy lesion distribution [4].

Coronary angiography is performed if the suspicion of acute coronary syndrome is intermediate or high, i.e., if at least one of the following elements is present: history of acute coronary syndrome, ECG with possible ischemia (not typical for pericarditis), unstable arrhythmias, marked elevation of troponin or rapid change in troponin, intermediate/high-risk GRACE score, or persistence of symptoms despite treatment for myopericarditis. A coronary CT angiography (or coronary CT scan) may be preferable in patients with intermediate risk.

In the vast majority of cases, the clinical course is spontaneously favorable, with initial conservative treatment consisting of anti-inflammatory drugs, optimal ionic control, and beta-blockers for their antiarrhythmic, myocardial protection, and afterload reduction effects. Severe cases, although rare, are managed according to standard protocols for acute heart failure. These include intensive care with inotropes and amines in case of cardiogenic shock, diuretics in case of vascular overload, and, once the acute situation has resolved, prescription of angiotensin-converting enzyme inhibitors that improve stunned myocardial contractility and left ventricular ejection fraction at one year [12,13]. Moreover, the non-negligible incidence of dilated cardiomyopathy secondary to myopericarditis at a distance should be noted.

The role of antibiotics remains to be determined, as most reported cases evolve favorably. In cases of clear common temporality, macrolides and quinolones cover the majority of *Campylobacter* spp., but it is necessary to adapt to local epidemiology, as quinolone resistance has been continuously increasing in the West since the late 1980s (probably related to the massive use of antibiotics in the agri-food industry). Currently, there is no recommended dosage or duration of treatment.

Finally, the importance of follow-up should not be overlooked, not only because there are reported cases of development of distant cardiomyopathy but also because a first case of relapse of *Campylobacter* myocarditis has recently been reported [14].

## Conclusions

Clinicians should consider myopericarditis in young patients presenting with chest pain following gastroenteritis. Thought to result from the synergistic effects of bacterial toxins and the immune response, the condition generally has a favorable prognosis but warrants hospitalization due to risks such as arrhythmia and heart failure. Further data are needed to confirm these hypotheses. Diagnosis relies on history (especially temporality between gastrointestinal and cardiac symptoms), ECG changes, blood/stool analysis, echocardiography, and cardiac MRI or CT in cases with low suspicion of ischemia. In moderate or high-risk situations, invasive coronary angiography may be warranted. Treatment includes non-steroidal anti-inflammatory drugs and colchicine, antibiotics when bacterial involvement is suspected, and beta-blockers if signs of acute heart failure are present. Follow-up is essential to monitor for relapse or secondary cardiomyopathy.
